# Trends and prescribing patterns of oral anti-neoplastic drugs: a retrospective longitudinal study

**DOI:** 10.3389/fpubh.2023.1294126

**Published:** 2023-11-23

**Authors:** Xiaoqun Lv, Weifang Ren, Shan Ran, Yuhan Zhao, Jihong Zhang, Jun Chen, Ning Zhang

**Affiliations:** ^1^Department of Pharmacy, Jinshan Hospital, Fudan University, Shanghai, China; ^2^Research Center for Clinical Medicine, Jinshan Hospital, Fudan University, Shanghai, China

**Keywords:** prescribing patterns, oral anti-neoplastic drugs, outpatients, DDDs, DDC, EGFR-TKI

## Abstract

**Background:**

Cancer as a global public health problem, imposes a heavy disease burden. With the rapid development of oral anti-neoplastic drugs, there has been a paradigm shift in the treatment of cancer from intravenous to oral administration.

**Objective:**

This study was conducted to investigate the trends and prescribing patterns of oral anti-neoplastic drugs in an academic tertiary hospital in China.

**Methods:**

A single-center and retrospective analysis was performed based on the prescriptions of outpatients treated with oral anti-neoplastic drugs from 2017 to 2022. Yearly prescriptions and expenditure were calculated according to their pharmacological classes, and trends were further analyzed. Defined daily doses (DDDs) and defined daily cost (DDC) of oral targeted anti-neoplastic drugs were also determined.

**Results:**

Both the number of prescriptions and expenditure of oral anti-neoplastic drugs increased progressively. There was a significant upward trend in the number and proportion of prescriptions for the older adult group, male group, and patients with gynecologic/genitourinary and respiratory cancer. Hormonal therapy agents accounted for the highest proportion of prescriptions, and letrozole was initially the most frequently prescribed drug. The number of DDDs of total oral targeted anti-neoplastic drugs showed a continuously ascending trend, primarily driven by the usage of epidermal growth factor receptor (EGFR) tyrosine kinase inhibitors (TKIs) and BCR-ABL TKIs.

**Conclusion:**

The prescriptions and expenditure of oral anti-neoplastic drugs, and the number of DDDs of oral targeted anti-neoplastic drugs all showed a progressively ascending trend. Further studies are needed to evaluate the long-term health and financial outcomes, and the factors influencing these prescribing patterns.

## 1 Introduction

Cancer is a substantial and global public health problem and is the second leading cause of death in the United States, contributing to a heavy burden of disease (1). Traditionally, the pharmacological therapy of cancer has predominantly been administered by intravenous route. However, oral anti-neoplastic drugs have experienced the highest rate of increase among all anticancer medications over the past 13 years (2). Due to the rapid growth in the use of oral anti-neoplastic drugs, there has been a paradigm shift in the treatment of cancer, moving from intravenous to oral administration.

Compared with parenteral therapy, oral anti-neoplastic drugs offer several advantages, such as improved flexibility, better quality of life, and the absence of complications induced by invasive administration (3). However, this breakthrough treatment shifts the responsibility of appropriate administration and monitoring from healthcare professionals to the patients (4). Although oral anti-neoplastic drugs are preferred by patients, they have created new challenges regarding the effectiveness and safety, including reduced healthcare visits, patient medication adherence, regular monitoring and management of adverse events, and the risk of drug–drug interactions with other medicines and foods (5–8). Furthermore, many oral anti-neoplastic drugs, especially the targeted therapy agents, are costly and impose an increased financial burden (9). The Chinese government has implemented policies to cope with increasing prices and improve accessibility (10, 11).

Given the shift of cancer treatment paradigm and the increasing clinical importance, the patterns and trends of oral anti-neoplastic drugs have been studied in some countries over the past decade, mainly in France and Manitoba (9, 12). However, limited studies regarding this issue have been conducted in China, specifically focusing on certain classes of anti-tumor drugs, such as EGFR-TKIs and human epidermal growth factor receptor 2 inhibitors (11, 13). Therefore, the present study aims to investigate the prescribing patterns and trends of oral anti-neoplastic drugs at our institution in China from 2017 to 2022, and analyze the DDDs and DDC values of oral targeted anti-neoplastic agents during this time period.

## 2 Materials and methods

### 2.1 Study design

This was a retrospective and observational study that reviewed the outpatient prescriptions of Jinshan Hospital, Fudan University in China from January 1, 2017 to December 31, 2022. It is an academic tertiary hospital with 1,000 regular hospital beds. Ethical approval was obtained from the Ethical Committee of Jinshan Hospital, Fudan University (JIEC 2023-S77). The study was designed as a retrospective research and the data was extracted from prescription records, so informed consent was waived. Due to the single-center study design, the study population may not be representative of the general population, leading to biased results.

### 2.2 Data source and study population

All prescriptions for oral anti-neoplastic drugs for outpatients diagnosed with cancer were identified and obtained from the hospital information system between 2017 and 2022. Patients treated with oral anti-neoplastic drugs for non-oncologic indications and prescriptions with incomplete information were excluded from the analysis. The following prescription information was collected: prescription code, prescription date, clinical department, sex and age of the patient, diagnosis, drug generic name, route of administration, usage, single dosage, unit price, total amount and expenditure of the prescribed drug.

In accordance with the National Comprehensive Cancer Network (NCCN) guidelines, the classes of oral anti-neoplastic drugs were further classified into three categories as follows: (1) chemotherapy agents (capecitabine, hydroxycarbamide, tegafur gimeracil and oteracil porassium, chlorambucil, methotrexate, doxifluridine), (2) targeted therapy agents (gefitinib, erlotinib, osimertinib, almonertinib, imatinib, dasatinib, flumatinib, apatinib, anlotinib, olaparib, pyrotinib), (3) endocrine therapy agents (abiraterone, bicalutamide, flutamide, letrozole, tamoxifen).

### 2.3 Assessment of drug use

The primary units of analysis were the prescriptions and expenditure of oral anti-neoplastic drugs. The total number of yearly prescriptions containing oral anti-neoplastic drugs were counted and the percentage of prescriptions was calculated. The total and yearly costs were calculated by aggregating the costs of all the prescriptions in Chinese Yuan (CNY). The overall trends were illustrated by the annual number of prescriptions and annual expenditure. For further analyses, the trends were stratified by age group, sex, cancer type, drug classification and specific drug. Age groups were classified as young (0–39 years), middle-aged (40–64 years), and older groups (≥65 years).

The defined daily dose (DDD) is a statistical unit used for calculating and comparing medicine consumption. As there is no standard DDD for anti-neoplastic drugs, the DDD was obtained based on the daily dose and indications from the instructions and authoritative specification databases as well as literatures (13, 14). The defined daily doses (DDDs) were calculated as the ratio of the total dose of a specific drug used in grams to the corresponding DDD value. Higher DDDs values indicate a higher frequency of prescribing the medicine (15). The defined daily cost (DDC), as a standardized measure of the cost of per DDD medicine, was measured at the prescription level and recorded in Yuan. DDC was calculated with the following formula: DDC = expenditure/(number of DDDs) (16).

### 2.4 Statistical analysis

Data were processed via Microsoft Access software. Descriptive statistics were applied to characterize baseline patient demographics, cancer type, and the consumption of oral anti-neoplastic drugs. Continuous variables were presented by mean values and standard deviation. The frequencies and percentages per category were used to describe the categorical variables. The trends in yearly number and expenditure of prescriptions were analyzed by the Mann–Kendall trend test, and the trends in proportions were assessed by the log-linear test. All statistical analyses were conducted in R (4.1.0) software. Statistical significance was set at *p* < 0.05. Sankey diagrams of EGFR-TKIs in 2022 were plotted with the R package alluvial. The other figures were made using Prism 5.0 (GraphPad Software).

## 3 Results

### 3.1 Overall trends in oral anti-neoplastic medication use and patient characteristics

**Figure 1:**
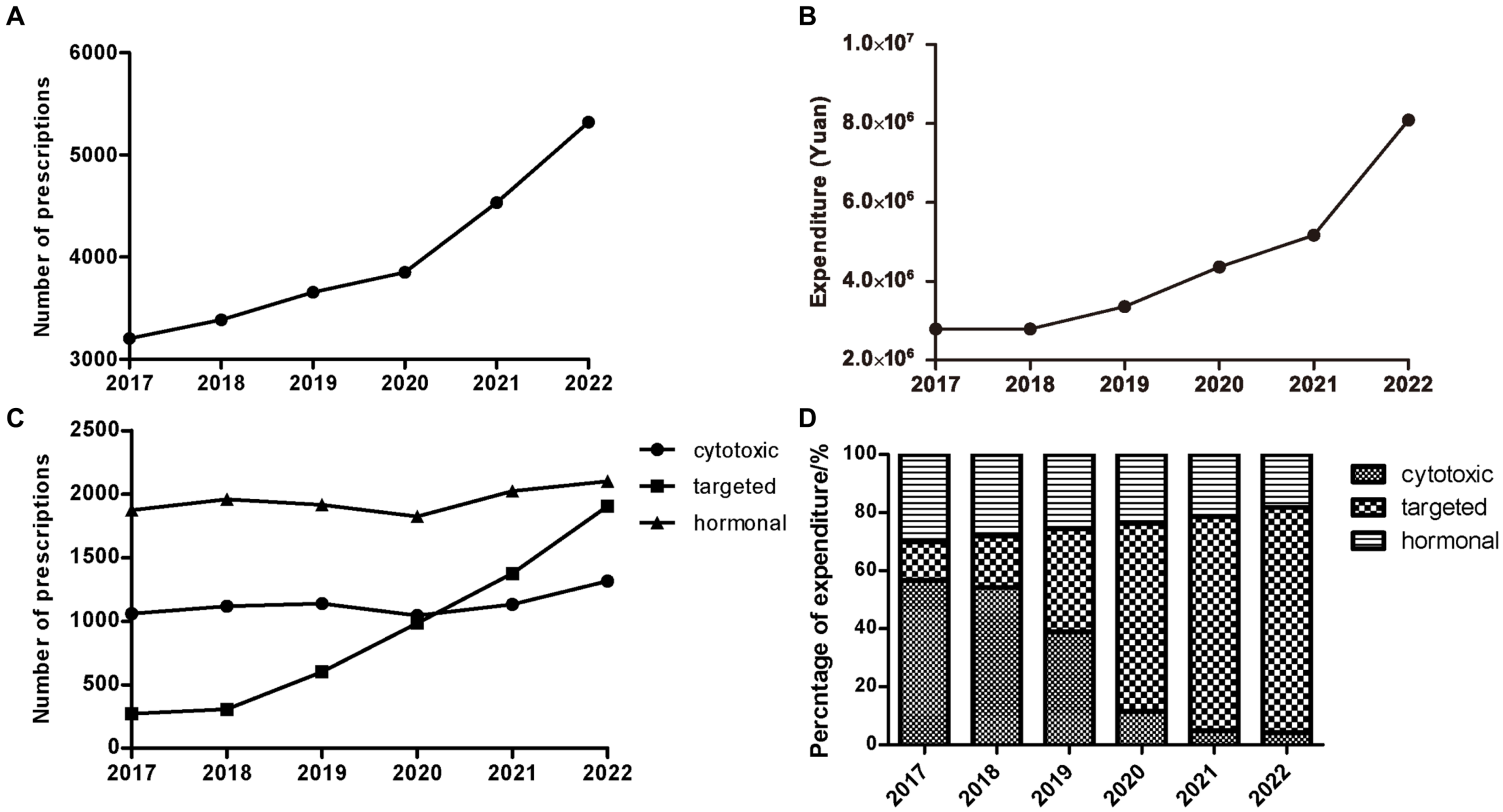
Trends of outpatients with treatment of oral anti-neoplastic agents from 2017 to 2022. **(A)** Trends of yearly total prescriptions. **(B)** Trends of yearly total expenditure. **(C)** Yearly prescriptions of different classes. **(D)** The percentage of yearly cost of specific drug classes.

Over the study period from 2017 to 2022, a total of 23,953 outpatient prescriptions meeting the inclusion criteria were included in the study. As shown in Figure 1A, the number of oral anti-neoplastic prescriptions increased progressively from 3,204 in 2017 to 5,322 in 2022, reflecting a 66.1% increase over the study period. The corresponding expenditure also continuously increased from 2,789,895 Chinese Yuan (CNY) in 2017 to 8,087,095 CNY in 2022 (Figure 1B). As Table 1 indicates, the average expenditure per prescription showed a significant increasing trend during the study period (*p* = 0.024).

**Table 1:** Demographic and clinical characteristics of cancer patients treated with oral anti-neoplastic drugs between 2017 and 2022.

Variables	Number of patients (%)							P_1_	P_2_
	2017	2018	2019	2020	2021	2022	Total		
Age, years, mean ± SD	63.51 ± 12.75	63.66 ± 12.96	65.96 ± 12.85	66.40 ± 12.97	67.10 ± 12.49	66.81 ± 12.89			
**Age group, *n* (%)**									
Young (0–39)	95 (2.97)	124 (3.66)	110 (3.01)	109 (2.83)	107 (2.36)	158 (2.97)	703 (2.94)	0.707	0.237
Middle-aged (40–64)	1,600 (49.94)	1,633 (48.23)	1,442 (39.45)	1,480 (38.42)	1,596 (35.20)	1,826 (34.31)	9,577 (39.98)	0.707	0.002
Older adult (≥65)	1,509 (47.10)	1,629 (48.11)	2,103 (57.54)	2,263 (58.75)	2,831 (62.44)	3,338 (62.72)	13,673 (57.08)	0.009	0.005
**Sex**									
Male	1,189 (37.11)	1,286 (37.98)	1,609 (44.02)	1,853 (48.10)	2,265 (49.96)	2,703 (50.79)	10,905 (45.53)	0.009	0.002
Female	2015 (62.89)	2,100 (62.02)	2,046 (55.98)	1,999 (51.90)	2,269 (50.04)	2,619 (49.21)	13,048 (54.47)	0.260	0.001
**Cancer type, *n* (%)**									
digestive tract	638 (19.91)	731 (21.59)	828 (22.65)	668 (17.34)	782 (17.25)	970 (18.23)	4,617 (19.28)	0.133	0.170
Respiratory	55 (1.72)	47 (1.39)	269 (7.36)	520 (13.50)	759 (16.74)	1,152 (21.65)	2,802 (11.70)	0.024	0.006
Breast	1,450 (45.26)	1,465 (43.27)	1,341 (36.69)	1,178 (30.58)	1,260 (27.79)	1,223 (22.98)	7,917 (33.05)	0.133	<0.001
Gynecologic/genitourinary	471 (14.70)	556 (16.42)	662 (18.11)	762 (19.78)	915 (20.18)	1,103 (20.73)	4,469 (18.66)	0.009	0.002
Hematology	569 (17.76)	577 (17.04)	548 (14.99)	683 (17.73)	743 (16.39)	811 (15.24)	3,931 (16.41)	0.060	0.292
Others	21 (0.66)	10 (0.30)	7 (0.19)	41 (1.06)	75 (1.65)	63 (1.18)	217 (0.90)	0.260	0.189
Total	3,204	3,386	3,655	3,852	4,534	5,322	23,953	0.009	
Average cost (CNY)	870.75	823.68	918.36	1132.13	1139.19	1519.56		0.024	

P_1_, *p*-value for trend in number of prescriptions, assessed by Mann–Kendall trend test; P_2_, *p*-value for trend in proportion of prescriptions, evaluated by log-linear analysis.

The characteristics of patients with oral anti-neoplastic prescriptions are presented in Table 1. The average age of the population showed continuous growth from 63.5 years in 2017 to 67.1 years in 2021, but slightly decreased to 66.8 years in 2022. The number of prescriptions was concentrated in middle-aged patients aged 40–64 years and older adult patients aged ≥65 years. In the middle-aged group, the number of prescriptions remained stable (*p* = 0.707) and the proportion continuously decreased over the 6-year period (*p* = 0.002). However, both the number and percentage of prescriptions for the older adult group patients increased progressively (all *p* < 0.01). There was a significantly upward trend in the number and proportion of prescriptions for male (all *p* < 0.01). Conversely, the female patients showed a reduction in the proportion of prescriptions (*p* = 0.001), while exhibiting no clear trend in the number (*p* = 0.26).

With respect to cancer type, patients receiving oral anti-neoplastic drugs were frequently diagnosed with digestive tract, respiratory, breast, gynecologic/genitourinary and hematology cancer. Both the number and proportion of prescriptions increased significantly in gynecologic/genitourinary and respiratory cancer (all *p* < 0.05), whereas the proportion decreased in breast cancer over the study period (*p* < 0.01).

### 3.2 Trends in prescriptions stratified by drug class and individual drug

A total of 22 oral anti-neoplastic drugs in three drug categories were involved in the study. In terms of medication category, hormonal therapy agents accounted for the highest proportion of the total prescriptions, ranging from 39.48 to 58.49% (Figure 1C; Table 2). As shown in Figures 1C and D, the number of hormonal and cytotoxic agent prescriptions remained stable during the study period, and the percentage of expenditure exhibited downwards trends in both drug classes. However, the rapid growth was observed for targeted agent class, where the number of oral drugs available increased from three to eleven. The use of targeted agents increased rapidly from 271 prescriptions in 2017 to 1905 prescriptions in 2022, and the corresponding cost significantly increased from 13.12 to 77.51% (all *p* < 0.05).

**Table 2:** Number of oral anti-neoplastic prescriptions from 2017 to 2022.

Medicine	Number of prescriptions (%)							P_1_	P_2_
	2017	2018	2019	2020	2021	2022	Total		
**Cytotoxic class**	1,059 (33.05)	1,118 (33.02)	1,139 (31.16)	1,044 (27.10)	1,132 (24.97)	1,316 (24.73)	6,808 (28.42)	0.260	0.002
Capecitabine	37 (1.15)	110 (3.25)	254 (6.95)	319 (8.28)	432 (9.53)	505 (9.49)	1,657 (6.92)	0.009	0.018
Hydroxycarbamide	278 (8.68)	289 (8.54)	301 (8.24)	349 (9.06)	383 (8.45)	379 (7.12)	1,979 (8.26)	0.024	0.210
Tegafur gimeracil and oteracil porassium	524 (16.35)	580 (17.13)	481 (13.16)	310 (8.05)	272 (6.00)	373 (7.01)	2,540 (10.60)	0.133	0.008
Chlorambucil, methotrexate, and doxifluridine	220 (6.87)	139 (4.11)	103 (2.82)	66 (1.71)	45 (0.99)	59 (1.11)	632 (2.64)	0.024	0.001
**Targeted class**	271 (8.46)	308 (9.10)	600 (16.42)	984 (25.55)	1,377 (30.37)	1,905 (35.79)	5,445 (22.73)	0.009	0.001
EGFR-TKIs	13 (0.41)	8 (0.24)	194 (5.31)	347 (9.01)	523 (11.54)	928 (17.44)	2,013 (8.40)	0.024	0.014
BCR-ABL TKIs	258 (8.05)	295 (8.71)	319 (8.73)	401 (10.41)	412 (9.09)	511 (9.60)	2,196 (9.17)	0.009	0.113
Apatinib, anlotinib, olaparib, and pyrotinib	0 (0.00)	5 (0.15)	87 (2.38)	236 (6.13)	442 (9.75)	466 (8.75)	1,236 (5.16)	0.008	0.056
**Hormonal class**	1,874 (58.49)	1,960 (57.89)	1,916 (52.42)	1,824 (47.35)	2,025 (44.66)	2,101 (39.48)	11,700 (48.85)	0.260	<0.001
Letrozole	1,103 (34.43)	1,140 (33.67)	1,129 (30.89)	918 (23.83)	967 (21.33)	888 (16.69)	6,145 (25.66)	0.133	0.001
Tamoxifen	339 (10.58)	307 (9.07)	177 (4.84)	191 (4.96)	212 (4.68)	200 (3.76)	1,426 (5.95)	0.452	0.008
Bicalutamide	334 (10.42)	381 (11.25)	477 (13.05)	584 (15.16)	608 (13.41)	626 (11.76)	3,010 (12.57)	0.009	0.306
Abiraterone and flutamide	98 (3.06)	132 (3.90)	133 (3.64)	131 (3.40)	238 (5.25)	387 (7.27)	1,119 (4.67)	0.060	0.031

EGFR-TKIs, gefitinib, erlotinib, osimertinib, and almonertinib.

BCR-ABL TKIs, imatinib, dasatinib, and flumatinib.

P_1_, *p*-value for trend in number of prescriptions, assessed by Mann–Kendall trend test; P_2_, *p*-value for trend in proportion of prescriptions, evaluated by log-linear analysis.

The annual number of prescriptions and expenditure for oral anti-neoplastic agents by category and individual drug are presented in Tables 2, 3. Letrozole was the most frequently prescribed drug at the beginning of the study. However, proportion of prescriptions containing letrozole gradually declined from 34.43% in 2017 to 16.69% in 2022 (*p* = 0.001), and the corresponding expenditure decreased from 15.43 to 1.56% (all *p* < 0.05). In contrast, the percentage of prescriptions for EGFR-TKIs increased dramatically from 0.24% in 2018 to 17.44% in 2022 (*p* = 0.014), and the corresponding cost also substantially rose from 2.45 to 43.73% (*p* = 0.012). Consequently, by the end of the study, EGFR-TKIs had become the most commonly prescribed drugs, with the highest expenditure.

**Table 3:** Cost of oral anti-neoplastic drugs from 2017 to 2022.

Medicine	Cost of Chinese yuan (%)							P_1_	P_2_
	2017	2018	2019	2020	2021	2022	Total		
**Cytotoxic class**	1,584,579 (56.80)	1,511,733 (54.20)	1,309,118 (39.00)	510,662 (11.71)	255,695 (4.95)	348,882 (4.31)	5,520,669 (20.79)	0.024	0.002
Capecitabine	27,724 (0.99)	74,766 (2.68)	159,188 (4.74)	179,518 (4.12)	115,736 (2.24)	120,759 (1.49)	677,691 (2.55)	0.260	0.818
Hydroxycarbamide	14,559 (0.52)	14,010 (0.50)	14,686 (0.44)	18,249 (0.42)	19,934 (0.39)	38,206 (0.47)	119,644 (0.45)	0.024	0.187
Tegafur gimeracil/oteracil porassium	1,450,872 (52.00)	1,374,210 (49.27)	1,094,723 (32.61)	290,648 (6.66)	113,203 (2.19)	179,125 (2.21)	4,502,781 (16.96)	0.024	0.003
Chlorambucil, methotrexate, and doxifluridine	91,424 (3.28)	48,747 (1.75)	40,521 (1.21)	22,247 (0.51)	6,822 (0.13)	10,792 (0.13)	220,553 (0.83)	0.024	<0.001
**Targeted class**	366,015 (13.12)	495,570 (17.77)	1,188,343 (35.40)	2,812,015 (64.48)	3,801,939 (73.61)	6,268,651 (77.51)	14,932,533 (56.25)	0.009	0.003
EGFR-TKIs	68,418 (2.45)	45,677 (1.64)	535,537 (15.95)	1,039,834 (23.84)	1,528,017 (29.58)	3,536,276 (43.73)	6,753,759 (25.44)	0.024	0.012
BCR-ABL TKIs	297,597 (10.67)	423,309 (15.18)	391,233 (11.66)	487,121 (11.17)	534,769 (10.35)	721,350 (8.92)	2,855,379 (10.76)	0.024	0.176
Apatinib, anlotinib, olaparib, and pyrotinib	0 (0.00)	26,584 (0.95)	261,573 (7.79)	1,088,808 (24.97)	1,724,705 (33.39)	1,873,119 (24.87)	5,323,395 (20.05)	0.008	0.064
**Hormonal class**	839,301 (30.08)	781,680 (28.03)	859,153 (25.60)	1,038,287 (23.81)	1,107,468 (21.44)	1,469,562 (18.17)	6,095,451 (22.96)	0.024	<0.001
Letrozole	430,374 (15.43)	414,166 (14.85)	389,613 (11.61)	334,802 (7.68)	102,512 (1.98)	125,783 (1.56)	1,797,250 (6.77)	0.024	0.006
Tamoxifen	9,115 (0.33)	7,120 (0.26)	36,991 (1.10)	48,020 (1.10)	54,488 (1.05)	64,876 (0.80)	220,610 (0.83)	0.024	0.115
Bicalutamide	364,249 (13.06)	318,797 (11.43)	389,251 (11.60)	563,784 (12.93)	442,840 (8.57)	360,276 (4.45)	2,439,197 (9.19)	0.707	0.059
Abiraterone and flutamide	35,563 (1.27)	41,597 (1.49)	43,298 (1.29)	91,681 (2.1)	507,628 (9.83)	918,627 (11.36)	1,638,394 (6.17)	0.008	0.017

EGFR-TKIs, gefitinib, erlotinib, osimertinib, and almonertinib.

BCR-ABL TKIs, imatinib, dasatinib, and flumatinib.

P_1_, *p*-value for trend in number of prescriptions, assessed by Mann–Kendall trend test; P_2_, *p*-value for trend in proportion of prescriptions, evaluated by log-linear analysis.

### 3.3 DDDs and DDC values of oral targeted anti-neoplastic agents

**Figure 2:**
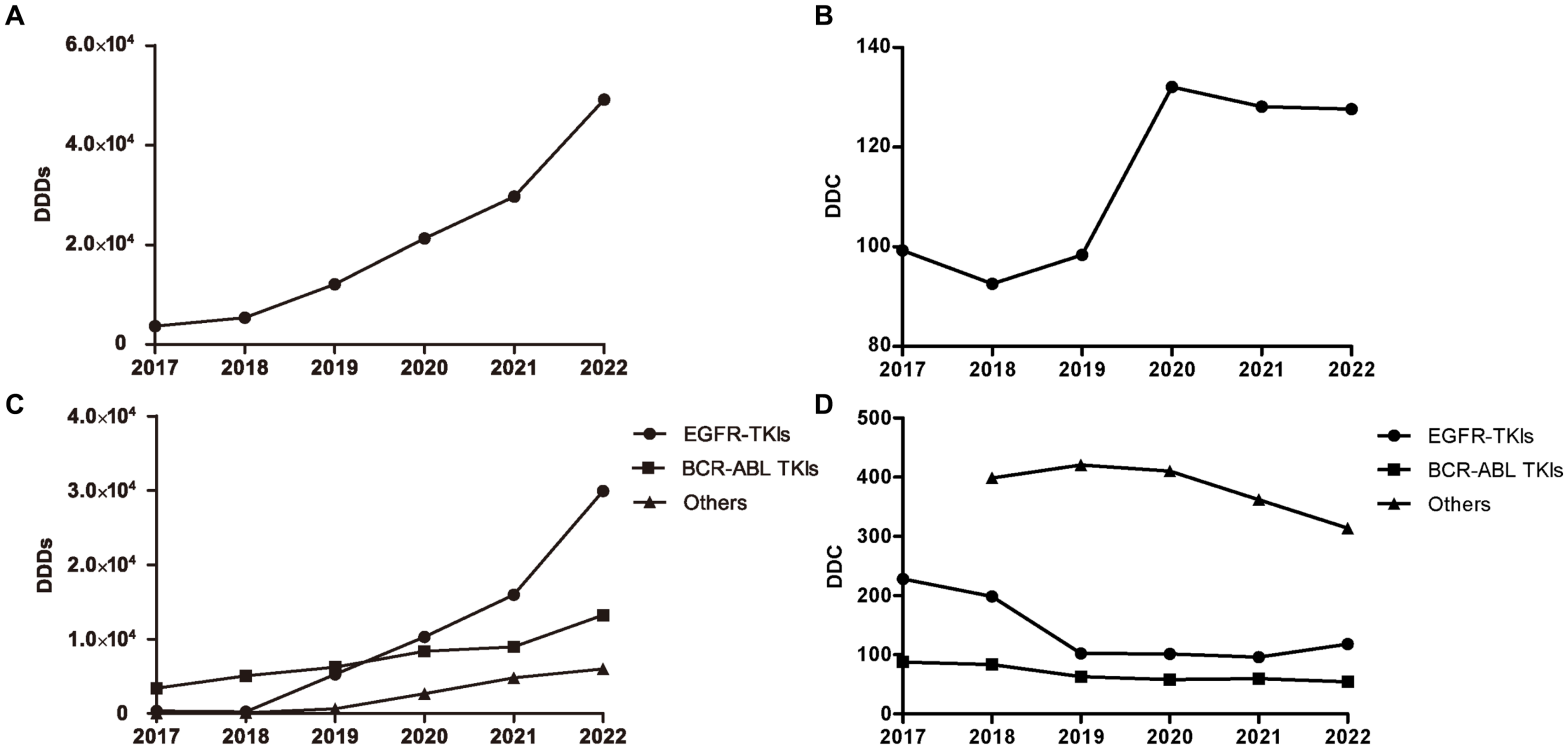
DDDs and DDC values of oral targeted anti-neoplastic drugs from 2017 to 2022. **(A)** DDDs of overall targeted anti-neoplastic drugs. **(B)** DDC of overall targeted anti-neoplastic drugs. **(C)** DDDs of three classes. **(D)** DDC of three classes. DDDs, defined daily doses; DDC, defined daily cost.

The number of DDDs of all oral targeted anti-neoplastic drugs exhibited a continuously ascending trend over the study period (Figure 2A). The average DDC of all targeted drugs changed slightly from 2017 to 2019 and from 2020 to 2022, but increased significantly from 2019 to 2020 due to the availability of olaparib and pyrotinib (Figure 2B).

As shown in Figure 2C, EGFR-TKIs and BCR-ABL TKIs were the most widely applied drugs, accounting for the majority of the total DDDs of targeted drugs from 2019 to 2022. The DDDs value of EGFR-TKIs increased dramatically from 2018 to 2022, and the DDDs value of BCR-ABL TKIs increased gradually since 2017. At the end of study, the number of DDDs of EGFR-TKIs was far more than that of other targeted drugs. However, the average DDC values of EGFR-TKIs and BCR-ABL TKIs were both at relatively low levels, and decreased year by year from 2017 to 2021 and 2017 to 2022, respectively. In addition, the DDC of the other targeted drugs all showed a descending trend (Figure 2D).

### 3.4 The consumption and distribution of EGFR-TKIs

**Figure 3:**
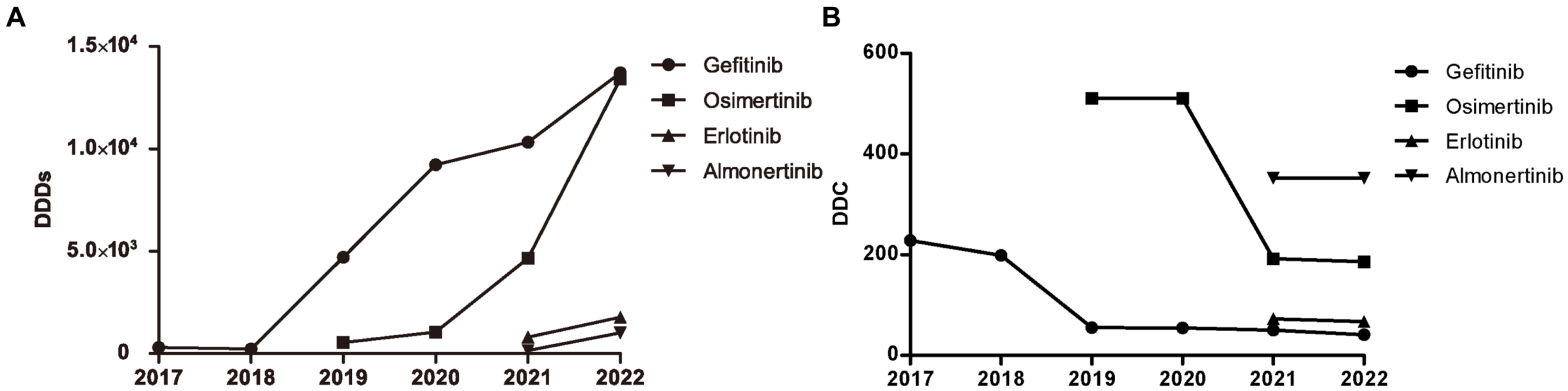
Consumption of four types of EGFR-TKIs from 2017 to 2022. **(A)** DDDs of four types of EGFR TKIs. **(B)** DDC of four types of EGFR TKIs. DDDs, defined daily doses; DDC, defined daily cost.

**Figure 4:**
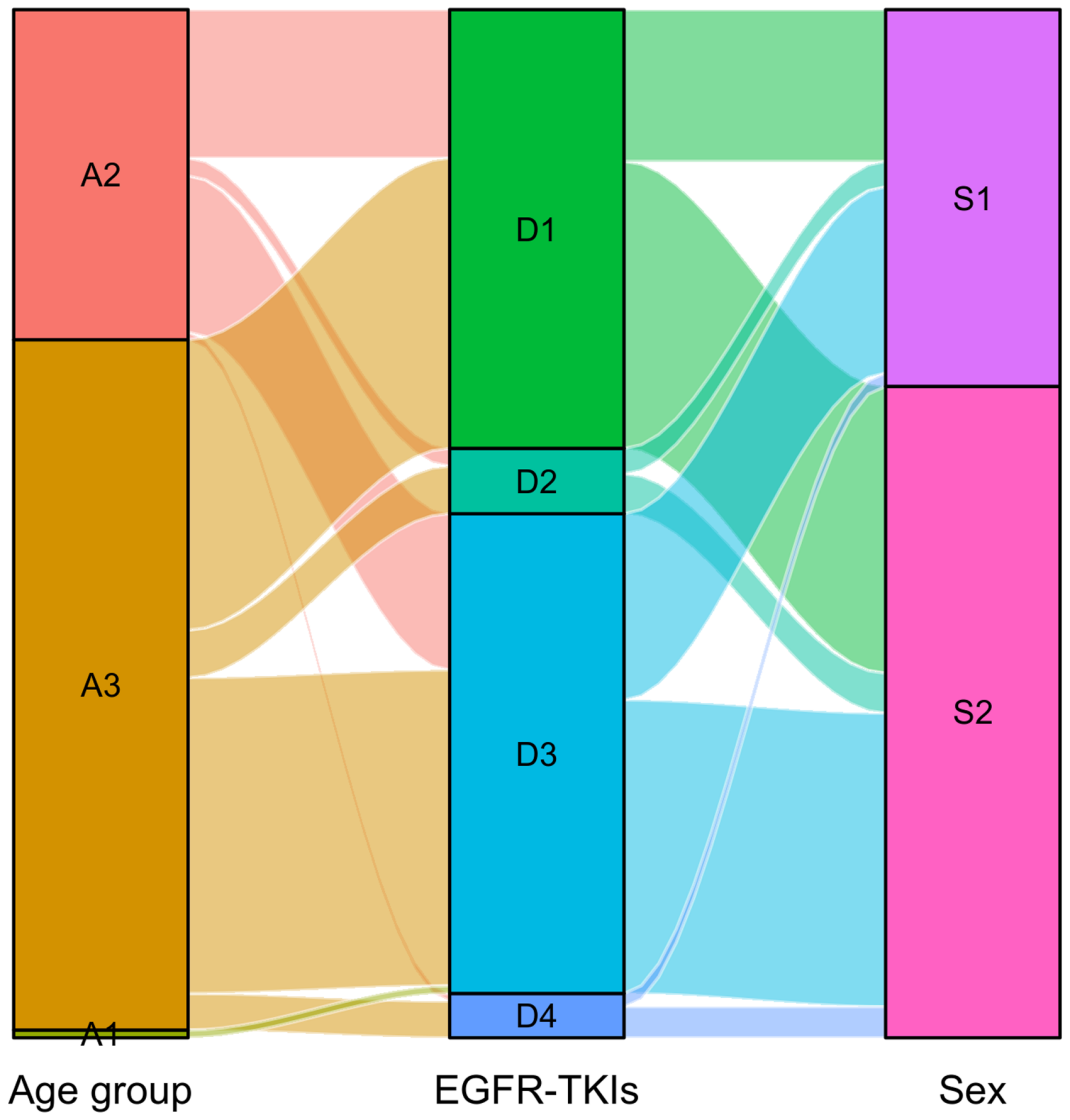
Sankey diagram of EGFR-TKIs in 2022. The age group and sex matched with the distribution of EGFR-TKIs. A1: Young, A2: Middle-aged, A3: Older group, D1: Gefitinib, D2: Erlotinib, D3: Osimertinib, D4: Almonertinib, S1:Male, S2: Female.

The distribution of DDDs and DDC values of drugs belonging to EGFR-TKIs in each year is shown in Figure 3, including gefitinib, erlotinib, osimertinib, and almonertinib. The number of DDDs of all four drugs increased dramatically, while the DDC of gefitinib and osimertinib decreased substantially in 2019 and 2021, respectively. The DDC of erlotinib and almonertinib remained relatively unchanged in 2021 and 2022. The Sankey diagram illustrated the relationship between age group, sex and the distribution of EGFR-TKIs in 2022 through lines, signifying the quantities via the line width (Figure 4).

## 4 Discussion

Oral anti-neoplastic therapy has emerged as a novel paradigm of anti-tumor treatment in recent years. The present study described the real-world patterns and trends regarding the use of oral anti-neoplastic drugs in a tertiary hospital in China over a 6-year period. Both the yearly prescriptions and corresponding expenditures of oral anti-neoplastic drugs increased progressively throughout the study period, particularly for oral targeted anticancer drugs. The DDDs of overall oral targeted anti-neoplastic drugs showed a continuously ascending trend over the study period, with EGFR-TKIs and BCR-ABL TKIs accounting for the largest proportion. In line with previous research conducted in other countries, this study found a significant increase of 66.1% in overall prescriptions and a 1.90-fold increase in expenditure (12, 17). A retrospective population-based study conducted in Manitoba demonstrated that the prevalence of oral anticancer agents use increased from 222 per 100,000 to 328 per 100,000, and the total cost of targeted oral anticancer agents per year for all cancer patients increased from $1.8 million to $19 million between the years 2003 and 2016 (12). Similarly, a retrospective analysis using survey data in France revealed that the proportion of cancer patients receiving oral anticancer treatments increased by four percentage points from 2004 to 2012, with an increase from 28.4 to 32.5% (17). This trend may reflect changes in cancer incidence and diagnosis patterns over time (12). Furthermore, the availability of oral anti-neoplastic drugs has significantly increased in recent years, leading to a shift toward the adoption of them as a therapeutic strategy for certain cancers (18). In terms of drug categories, the prescriptions of oral targeted anti-neoplastic drugs showed a significant increase during the study period. The rapid increase in prescriptions for targeted agents can be attributed to several factors. Firstly, with the advancements in medical technology and research, FDA-approved targeted agents have experienced a remarkable surge in the past 20 years and have become mainstream cancer treatments (19). Secondly, targeted drugs have shown high potency, reduced toxicity and increased survival rates compared to traditional chemotherapy drugs. This has resulted in a growing interest and demand for these treatment options among healthcare providers and patients. Thirdly, the accessibility and affordability of targeted anti-cancer drugs have significantly increased following the implementation of government healthcare policies (20, 21). The prescriptions of oral cytotoxic and hormonal anti-neoplastic drugs remained stable, resulting in decreasing proportions.

In this study, the average age of the population increased gradually from 2017 to 2021, and both the number and percentage of prescriptions rose progressively in older adult patients aged ≥65 years. These results may be attributed to increased life expectancy, longer survival of patients and preference of clinicians for oral anticancer drugs instead of parenteral therapy in older adult patients to improve their quality of life (9). The absence of social support, limited access to care, and financial constraints may serve as major obstacles for older patients in utilization of oral anticancer drugs (22).

In the first few years of this study, the number of prescriptions for females was dramatically greater than those for males due to the high proportion of letrozole. Letrozole is known as a potent third-generation aromatase inhibitor and has been widely used in the adjuvant, neoadjuvant, and metastatic therapy of hormone receptor-positive breast cancer (23). However, with the widespread application of oral targeted anticancer drugs in clinical practice, the disparity in proportion between males and females has gradually decreased.

Regarding oral targeted anti-neoplastic drugs, the number of DDDs showed an upward trend, while the number of DDC showed a downward trend. DDC is an efficient indicator that guides the market prices of pharmaceutical products (15). Consistently, previous studies have indicated that drug price was an important determinant of drug consumption, and there was a negative relationship between DDC and the number of DDDs (11, 13, 24). In order to improve the availability and affordability of drugs, China established its fundamental health insurance system in 2009, which was enhanced by the implementation of price negotiation and mandatory reimbursement policies in 2017 (13). The policies of price negotiation have been implemented in many countries, which resulted in a significant reduction in drug prices and an increase in drug consumption (24, 25). The Chinese government has implemented six rounds of national drug price negotiations since 2016 to include innovative and expensive drugs, particularly anticancer drugs, in the national list of reimbursable medicines. The average price discount for newly-added drugs has exceeded 50% from 2018 to 2021 (26). Furthermore, national centralized drug procurement organized by the Chinese government has effectively reduced the price of bid-winning anti-cancer drugs (27). National volume-based procurement was launched in 2018 to negotiate drug price with manufacturers in “4 + 7” pilot cities, which covered 4 provincial municipalities and 7 sub-provincial cities (10). The procurement volume was set based on 60–70% of the actual annual drug consumption of all public hospitals in pilot cities in the previous year (21). The centrally purchased pilot drugs were high-quality generic drugs with the same therapeutic effect through the quality consistency evaluation procedure. Most of them were drugs for the treatment of chronic diseases, and major diseases such as tumors.

In this study, the DDDs value of EGFR-TKIs increased dramatically from 2018 to 2022, far exceeding that of other targeted drugs at the end of the study. The latest monitoring data in 2023 showed that lung cancer had been the leading cancer in Shanghai. EGFR-TKIs have been used as a novel therapeutic strategy in patients with advanced non-small cell lung cancer (NSCLC) with EGFR mutations, significantly improving prognosis. Although the first generation EGFR-TKIs, including gefitinib and erlotinib, have shown high efficacy, resistance has emerged due to T790M mutation (28). Osimertinib, a third-generation and central nervous system-active EGFR-TKI targeting T790M mutation, has exhibited longer progression-free survival (PFS) compared to the first generation EGFR-TKIs (29). Almonertinib, another third-generation EGFR-TKI, was approved by China in March 2020 as a novel treatment option for EGFR T790M+ NSCLC (30). Our data showed a marked increase in the number of DDDs for gefitinib and osertinib, far exceeding erlotinib and almonertinib. Geftinib and erlotinib have been included in the national health insurance in 2017, and osimertinib in 2019. Additionally, multiple rounds of price negotiations have been conducted for EGFR-TKIs, resulting in a decrease in DDC and improved availability (11).

The results of the present study showed that both the number of BCR-ABL TKIs prescriptions and expenditure significantly increased over the study period. Moreover, the DDDs value of BCR-ABL TKIs has been gradually increasing since 2017, accompanied by a gradual decline in DDC. BCR-ABL TKIs are the first-line treatment for patients with chronic myelocytic leukemia (CML) and Ph chromosome-positive acute lymphoblastic leukemia (31). With the development of BCR-ABL TKIs including first-generation imatinib and second-generation dasatinib, most CML patients experience long-term remissions and have a life expectancy close to normal (32). Flumatinib, a novel second-generation BCR-ABL TKI, has been launched in the Chinese market with an optimized structure based on imatinib to achieve higher potency (33).

There were certain limitations in our study. First, it was a single-center and retrospective design, which restricted the generalizability of the data to other populations or settings. Second, the term consumption referred to the quantity of prescribed drugs rather than administered drugs, due to the lack of information about medication compliance. Third, our analysis was carried out based on prescription data, and lacked the clinical information such as laboratory tests and biological characteristics, which limited the ability to assess the appropriateness of drug prescriptions and evaluate factors influencing prescribing patterns. Furthermore, while the study focused on prescription trends and drug consumption, it does not provide insights into patient outcomes or the effectiveness of the prescribed treatments.

## 5 Conclusion

In this study, the use of oral anti-neoplastic drugs has been increasing annually, especially oral targeted anticancer drugs. Among them, EGFR-TKIs and BCR-ABL TKIs accounted for the majority in terms of defined daily doses (DDDs), but with relatively low levels of DDC. Further studies are warranted to explore the factors influencing the prescribing patterns and to conduct the cost-effectiveness analysis of oral anti-neoplastic treatments.

## Data availability statement

The raw data supporting the conclusions of this article will be made available by the authors, without undue reservation.

## Ethics statement

The studies involving humans were approved by Ethics Committee of Jinshan Hospital, Fudan University. The studies were conducted in accordance with the local legislation and institutional requirements. Written informed consent for participation was not required from the participants or the participants’ legal guardians/next of kin in accordance with the national legislation and institutional requirements.

## Author contributions

XL: Conceptualization, Funding acquisition, Investigation, Writing – original draft, Writing – review & editing. WR: Data curation, Resources, Validation, Writing – original draft. SR: Methodology, Project administration, Writing – original draft. YZ: Data curation, Project administration, Writing – original draft. JZ: Methodology, Software, Writing – original draft. JC: Formal analysis, Software, Visualization, Writing – review & editing. NZ: Conceptualization, Resources, Supervision, Validation, Writing – review & editing.
